# Evaluating GPT-5 for Melanoma Detection Using Dermoscopic Images

**DOI:** 10.3390/diagnostics15233052

**Published:** 2025-11-29

**Authors:** Qingguo Wang, Ihunna Amugo, Harshana Rajakaruna, Maria Johnson Irudayam, Hua Xie, Anil Shanker, Samuel E. Adunyah

**Affiliations:** 1 Department of Biochemistry, Cancer Biology, Neurosciences and Pharmacology, School of Medicine, Meharry Medical College, Nashville, TN 37208, USA; 2 Department of Oral Diagnostic Sciences and Research, School of Dentistry, Meharry Medical College, Nashville, TN 37208, USA; iamugo23@mmc.edu (I.A.);; 3 The Office for Research and Innovation, Meharry Medical College, Nashville, TN 37208, USA

**Keywords:** melanoma diagnosis, dermoscopy, large language model, ChatGPT, GPT-5

## Abstract

**Background**: Melanoma is the deadliest form of skin cancer, for which early detection is crucial and can lead to positive survival outcomes. Advances in AI, particularly large language models (LLMs) such as GPT-5, present promising opportunities to support melanoma early detection, but their performance in this domain has not been systematically assessed. **Objectives**: Assess GPT-5’s diagnostic performance on dermoscopic images. **Methods**: GPT-5 was evaluated on two public benchmark datasets: the ISIC Archive and HAM10K, using 100 and 500 randomly selected dermoscopic images, respectively. Via the OpenAI Application Programming Interface (API), GPT-5 was prompted to perform three tasks: (1) top-1 or primary diagnosis, (2) top-3 differential diagnoses, and (3) malignancy discrimination (melanoma vs. benign). Model outputs were compared with histopathology-verified ground truth, and performance was measured by sensitivity, specificity, accuracy, F1 score, and other metrics. **Results**: GPT-5 achieved modest accuracy in top-1 or primary diagnosis but markedly improved performance in top-3 differential diagnoses, with sensitivity > 93%, specificity > 86%, accuracy ≥ 92%, and F1 score > 91%. For malignancy discrimination, GPT-5 showed more balanced sensitivity and specificity than GPT-4-based models (GPT-4V, GPT-4T, and GPT-4o), resulting in more reliable classification overall. **Conclusions**: GPT-5 outperformed GPT-4 and its derivatives, particularly in differential diagnosis, highlighting its potential for clinical decision support and medical education. However, GPT-5 also showed a tendency to misclassify melanoma as benign, underscoring the need for cautious clinical interpretation and refinement.

## 1. Introduction

Melanoma is the deadliest form of skin cancer, accounting for nearly 75% of skin cancer deaths [1]. According to the Melanoma Facts & Figures published by the American Cancer Society, an estimated 104,960 new cases will be diagnosed, and 8430 people will die of this disease in the U.S. in 2025 [2]. Although melanoma is highly lethal, early detection offers an exceptional opportunity for favorable outcomes, with five-year survival rates approaching 99% and the potential for cure.

With the shortage of experienced dermatologists in many regions and the growing demand for accessible, accurate, and cost-effective diagnostic resources, innovative approaches for early melanoma detection are urgently required. Large language model (LLM)–based chatbots, such as ChatGPT [3], have recently emerged as promising tools to support melanoma detection. Although not originally designed for healthcare, these systems can generate human-like responses with notable accuracy across a wide range of medical topics [4,5,6,7,8,9,10], offering new opportunities for disease surveillance, biomedical research, and education. More recently, advanced multimodal LLMs (MLLMs) [3,11,12], such as the newly released Generative Pre-trained Transformer 5 (GPT-5), extend these capabilities by integrating visual and auditory inputs with textual data, substantially broadening their potential utility in clinical decision support and public health.

Compared with traditional resources, LLMs offer distinct advantages for diagnostic support, including lower cost, continuous availability, good accuracy for many diseases and conditions, customizable interactions, and user-friendly interfaces. As a result, people increasingly turn to them for medication information, self-diagnosis, and disease-prevention guidance [13,14,15,16,17]. Medical students and clinicians also use them to acquire knowledge and support clinical decision-making [15,18,19].

Numerous studies have investigated the use of LLMs for melanoma detection, biomedical research, and education [20,21,22,23]. Shifai et al. evaluated GPT-4 Vision (GPT-4V) on 100 dermoscopic images from the ISIC Archive and reported performance below that of market-approved AI algorithms [20]. Cirone et al. assessed GPT-4V and the Large Language and Vision Assistant (LLaVA) for melanoma detection and found that both models were able to distinguish between benign skin lesions and melanoma [22]. Pillai et al. further evaluated GPT-4V across nine common dermatologic conditions, demonstrating that diagnostic accuracy improved substantially when clinical photographs were combined with textual inputs compared with image-only assessments [24]. More recently, Sattler et al. examined GPT-4 Turbo (GPT-4T) and GPT-4 Omni (GPT-4o) using the publicly available HAM10K dataset, reporting classification sensitivities of 76.3% and 96.8%, specificities of 32.9% and 18.4%, and accuracies of 54.6% and 57.7%, respectively [21].

Beyond LLM-based approaches, a substantial body of research in machine learning (ML) and deep learning (DL) has focused on melanoma detection. Convolutional neural network (CNN)–based models, including advanced architectures like Inception-v4, ResNet, DenseNet, and EfficientNet, have demonstrated dermatologist-level performance in landmark studies and ISIC Challenges [25,26,27,28]. Furthermore, this technology has moved into clinical settings, with AI-assisted tools such as FotoFinder’s Moleanalyzer-Pro and DermaSensor’s spectroscopy-based device incorporating ML to aid dermoscopic assessment [29,30]. These advances illustrate the rapidly evolving landscape of AI in dermatology and underscore the importance of evaluating new multimodal models, such as GPT-5, within this extensive technological context.

Despite these advances, important gaps remain. Most prior studies relied on relatively small and homogeneous datasets, which limited the generalizability of their findings. In addition, they often focused on a single diagnostic task, such as malignancy discrimination, rather than providing a comprehensive evaluation across multiple tasks. Furthermore, with the continual iteration and rapid improvement of LLMs, earlier assessments may not accurately reflect the capabilities of newer models such as GPT-5, released in August 2025, whose performance in melanoma detection has not yet been systematically evaluated.

Given the increasing use of LLMs for self-examination and clinical applications, rigorous evaluation in high-stakes contexts is essential to establish quality-control mechanisms, mitigate risks of inaccurate outputs, and guide their safe integration into melanoma care. To address this need, the present study evaluates the newly released GPT-5 for melanoma detection.

## 2. Materials and Methods

Among the modalities available for melanoma detection, such as reflectance confocal microscopy [31], histopathological examination [32], and emerging non-invasive techniques (e.g., total body photography and teledermatology) [33,34], dermoscopy remains the most widely adopted and clinically validated [35]. Given its central role in dermatologic practice and the availability of large, well-annotated public datasets [36,37], we employ dermoscopic imaging to evaluate the performance of LLMs in melanoma detection.

### 2.1. Data Sources

The dermoscopic images used for assessment were drawn from two widely used, publicly accessible repositories of histopathology-verified skin lesion images, the International Skin Imaging Collaboration (ISIC) Archive and the Human Against Machine with 10,000 training images (HAM10000 or HAM10K) [36,37]. From the ISIC Archive, 100 dermoscopic images previously included in a benchmark study were selected [20]. For each case, both the dermoscopic image and accompanying metadata were retrieved from the ISIC, including clinical diagnosis, anatomical site, Breslow thickness, and patient characteristics such as age and sex. For consistency, lesions labeled as melanoma were grouped as malignant, while nevi, keratosis-like lesions, dermatofibromas, and vascular lesions were grouped as benign.

The HAM10K dataset contains 10,015 dermoscopic images, released as a training set for academic machine learning purposes and made publicly available through the ISIC Archive [36]. From HAM10K, we randomly selected 250 melanoma and 250 benign cases to form a balanced evaluation set. The canonical diagnosis provided with each image was used to establish ground-truth labels for model evaluation.

### 2.2. Diagnostic Tasks and Study Design

Although many LLMs are publicly available, we focused our evaluation on GPT-5, a leading model launched by OpenAI in August 2025, to ensure comparability with other major LLM-based benchmarks in the field [20,21,38].

GPT-5’s detection performance was evaluated across three related tasks: (1) **top-1 diagnosis**, defined as the model’s highest-ranked (primary) prediction; (2) **top-3 differential diagnosis**, the ordered list of the three diagnoses the model ranked as most likely; and (3) **malignancy discrimination**, GPT-5’s binary classification of lesions as malignant or benign. A case was considered correct for the top-1 task only if the model’s primary prediction matched the ground truth, and correct for the top-3 task if the true diagnosis appeared anywhere within the model’s top three predictions. Together, these tasks align with common LLM evaluation practices and mirror elements of clinical reasoning.

**Figure 1:**
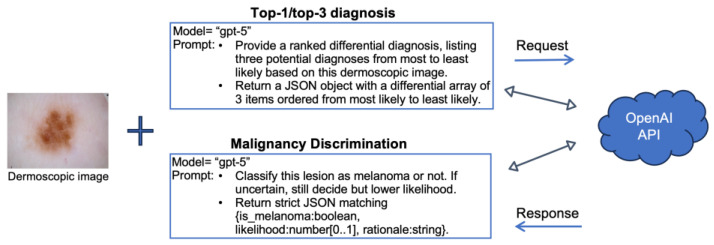
Our study design: dermoscopic images were input to GPT-5 via the OpenAI API for top-1 or primary diagnosis, top-3 diagnoses, or malignancy discrimination. The example image (ISIC_0021052) is from the ISIC Archive; prompts shown in text boxes were reformatted for clarity.

Because of the data set size and the large number of assessments required, manual submission of images through the default ChatGPT interface is not feasible. Therefore, we accessed GPT-5 programmatically through the OpenAI Application Programming Interface (API). Figure 1 illustrates our overall study design. The model was used “as is,” without any fine-tuning or external training, to reflect real-world deployment. Dermoscopic images were submitted to GPT-5 with standardized prompts, which consisted of two components: (1) an instruction specifying the diagnostic task, and (2) a formatting instruction requesting output in JSON (JavaScript Object Notation) format for standardized downstream analysis. Following the approach described by Shifai et al. [20], we did not query GPT-5 separately for top-1 diagnosis; rather, the model’s single highest-ranked prediction from the top-3 output was used to evaluate top-1 diagnostic performance.

### 2.3. Performance Metrics and Implementation

Model responses were parsed, stored, and compared against ground-truth clinical diagnoses to calculate sensitivity, specificity, accuracy, F1 score, and other performance metrics. The analysis pipeline was implemented using the Python (v3.12.7) programming language, and visualizations were generated in R using the ggplot2 package (v4.3.3 (2024-02-29)). The Python scripts developed for this study, including code for downloading and processing images from the ISIC Archive and HAM10K, as well as code for querying the OpenAI API, are publicly available at https://github.com/qwangmsk/Melanoma-Detect (accessed on 27 November 2025).

## 3. Results

### 3.1. Evaluating GPT-5 on the ISIC Data

**Figure 2:**
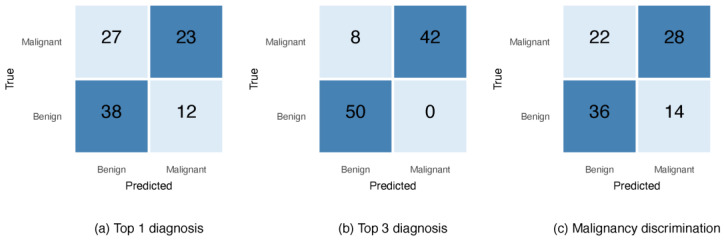
Confusion matrices of GPT-5 detection performance on 100 ISIC images. Panels show results for (**a**) top-1 diagnosis, reflecting the model’s primary prediction, (**b**) top-3 diagnosis, counted as correct when the ground truth appears within the three highest-ranked outputs, and (**c**) malignancy discrimination, GPT-5’s binary classification of lesions as malignant or benign.

For each of the 100 ISIC dermoscopic images, GPT-5’s predictions were compared with the reference clinical diagnosis, and performance was summarized in confusion matrices (Figure 2). As shown in Figure 2, GPT-5’s misclassifications were asymmetrical, with a greater tendency to classify malignant lesions as benign across the three diagnostic tasks. In the top-1 diagnostic setting (Figure 2a), 27 melanomas were incorrectly predicted as benign, compared with 12 benign lesions misclassified as malignant. Although diagnostic performance improved when considering the top-3 differential diagnoses (Figure 2b) and malignancy discrimination (Figure 2c), this underlying bias persisted. This raises concern in applying GPT-5 to melanoma diagnoses, given the higher risk associated with false negatives.

In quantitative terms, GPT-5 achieved a sensitivity of 46.0%, specificity of 76.0%, accuracy of 61.0%, and an F1 score of 54.0% for top-1 diagnosis. Performance improved substantially in the top-3 differential setting, with 100% sensitivity, 86.2% specificity, 92.0% accuracy, and an F1 score of 91.3%. For malignancy discrimination, performance was intermediate, with 56.0% sensitivity, 72.0% specificity, 64.0% accuracy, and an F1 score of 60.9%. These results demonstrate that while GPT-5’s single best prediction remains limited, its broader differential diagnostic ability provides far stronger clinical support.

### 3.2. Comparing GPT-5 with Other Studies on the ISIC Data

**Figure 3:**
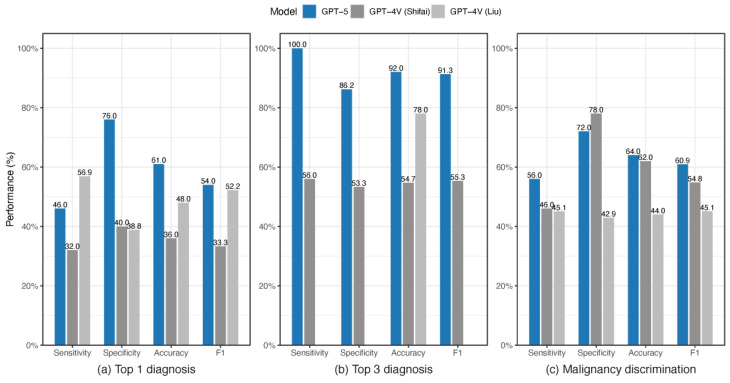
Detection performance of GPT-5 compared with prior GPT-4V models (Shifai et al. [20] and Liu et al. [38]) on ISIC images across three tasks: top-1 diagnosis, top-3 differential diagnoses, and malignancy discrimination.

Table 1 and Figure 3 compare our findings with two prior studies by Shifai et al. [20] and Liu et al. [38] that evaluated GPT-4V, an earlier version of ChatGPT. To allow direct comparison, we aligned our study design with these investigations, both of which used 100 ISIC dermoscopic images. The dataset used by Shifai et al. was identical to ours, and we employed the same prompt when querying GPT-5 (shown in Figure 1) to ensure consistency across studies.

As shown in Table 1 and Figure 3, GPT-5 outperformed GPT-4V across the three diagnostic tasks. For top-1 diagnosis, GPT-5 achieved higher accuracy (61.0% vs. 36.0–48.0%) and F1 score (54.0% vs. 33.3–52.2%), indicating improved reliability in primary prediction. The advantage became more pronounced in the top-3 differential diagnosis task, where GPT-5’s accuracy of 92.0% and F1 score of 91.3% significantly surpassed GPT-4V’s 54.7–78.0% accuracy and 55.3% F1. For malignancy discrimination, GPT-5 also exceeded GPT-4V, with an accuracy of 64.0% and an F1 score of 60.9% compared to 44.0–62.0% accuracy and 45.1–54.8% F1 of GPT-4V. Collectively, these results suggest substantial diagnostic improvements of GPT-5 over GPT-4V, particularly in top-3 differential diagnoses.

It is worth noting that several other studies also evaluated GPT-4V (and GPT-4) [23,39]. We did not include them in our comparison for two reasons: (1) their findings were broadly consistent with those of the two studies already cited, and (2) they employed different imaging datasets for benchmark testing, making their results not directly comparable with those presented here.

### 3.3. Evaluating GPT-5 on the HAM10K Data

**Figure A1:**
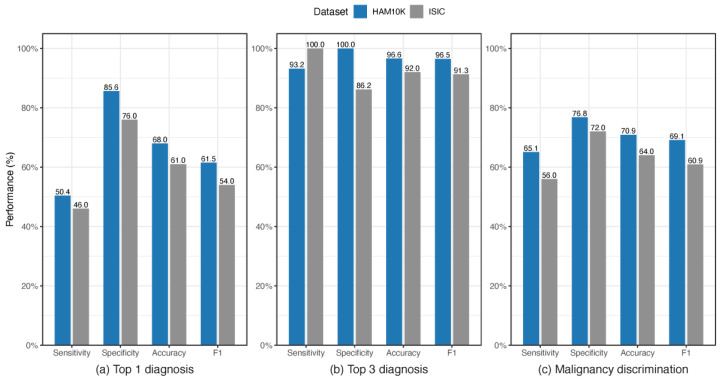
Comparison of GPT-5 detection performance for melanoma on ISIC and HAM10K datasets.

GPT-5’s diagnostic capability on the HAM10K data is summarized in Table 2. For the top-1 diagnosis task, the model achieved a sensitivity of 50.4%, specificity of 85.6%, accuracy of 68.0%, and an F1 score of 61.5%, consistently higher than its performance on the ISIC data (Figure A1 in Appendix A.1). As observed with the ISIC data, performance improved markedly when considering the top-3 differential diagnoses on HAM10K, with sensitivity increasing to 93.2%, specificity to 100%, accuracy to 96.6%, and the F1 score to 96.5%. In the malignancy discrimination task, GPT-5 achieved a sensitivity of 65.1%, specificity of 76.8%, accuracy of 70.9%, and an F1 score of 69.1%. Overall, GPT-5 demonstrated stronger performance across all three diagnostic tasks on HAM10K compared with ISIC (Figure A1 in Appendix A.1).

**Figure A2:**
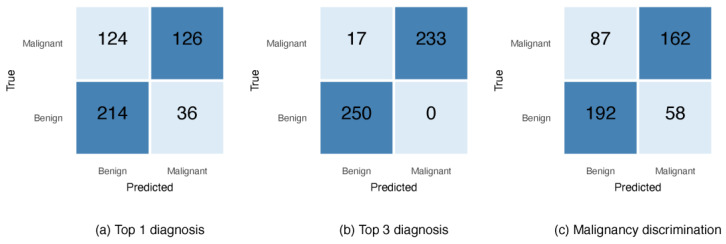
Confusion matrices of GPT-5 detection performance on HAM10K images.

Additionally, analysis of misclassifications revealed the same pattern on HAM10K: GPT-5 showed a greater tendency to classify malignant lesions as benign, a bias observed in all three diagnostic tasks (Figure A2 in Appendix A.2).

### 3.4. Comparing GPT-5 with Another Study on HAM10K

Next, we extended our comparison by evaluating GPT-5 against its more recent predecessors, GPT-4o and GPT-4T, whose performance was assessed by Sattler et al. recently on 500 dermoscopic images randomly selected from the HAM10K dataset [21]. Because Sattler et al. did not provide the identifiers of their images, we replicated their sampling strategy by randomly selecting 250 melanoma and 250 benign cases from HAM10K, as described in Section Section 2.1 earlier. As our sampling was conducted independently, the overlap between the two sets is expected to be small, which may influence the comparability of the results.

**Figure 4:**
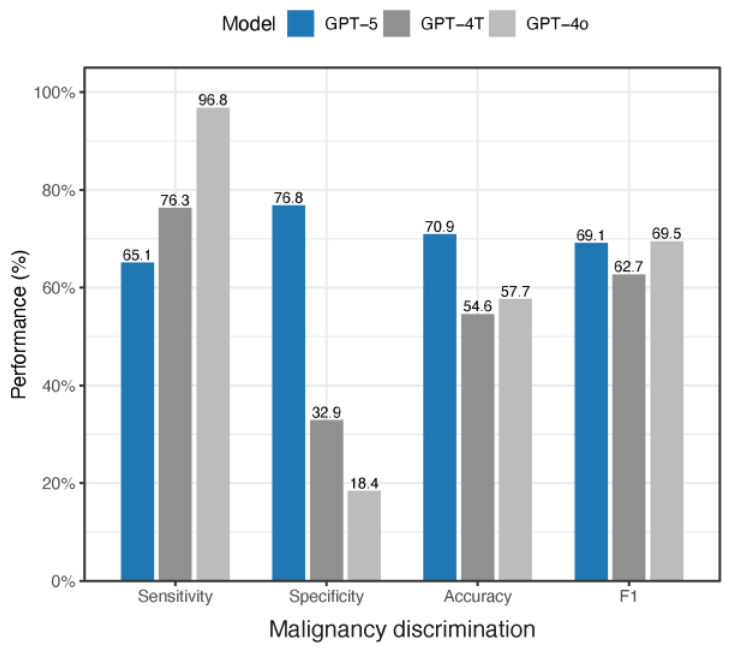
Comparison of GPT-5, GPT-4T, and GPT-4o performance in melanoma malignancy discrimination on 500 HAM10K images.

GPT-5’s predecessors, GPT-4o and GPT-4T, were evaluated by Sattler et al. only for malignancy discrimination on HAM10K [21], as provided in Table 2 and Figure 4. Compared with these two models, GPT-5 demonstrated a more balanced performance. GPT-4T achieved higher sensitivity (76.3%) but much lower specificity (32.9%), resulting in reduced accuracy (54.6%) and an F1 score of 62.7%. GPT-4o reached even higher sensitivity (96.8%) but at the cost of very poor specificity (18.4%), yielding an accuracy of 57.7% and an F1 score of 69.5%. In contrast, GPT-5 provided a more favorable balance between sensitivity and specificity, offering more reliable malignancy discrimination. When comparing the F1 score, GPT-5 did not show improvement over its immediate predecessor, GPT-4o. The observed differences in performance here should be interpreted cautiously, however, as our evaluation was conducted on a different randomly selected subset of HAM10K images, which may limit direct comparability with GPT-4o and GPT-4T.

Sattler et al. also used an additional 1000 randomly selected HAM10K images to assess the effect of prompting on GPT-4o. As GPT-5 is less sensitive to prompt variation than GPT-4 and its derivatives, we did not repeat this assessment.

### 3.5. Agreement Beyond Chance and Threshold-Independent Performance

**Figure A3:**
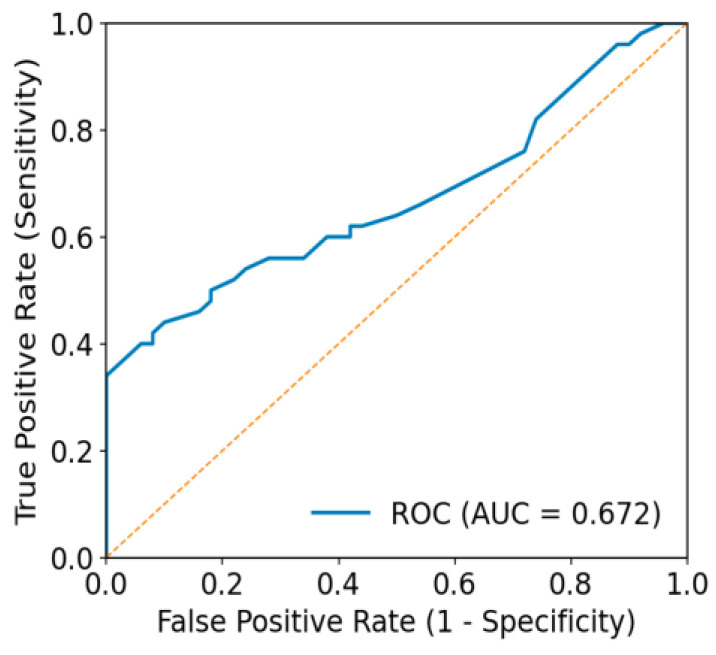
Receiver operating characteristic (ROC) curve for GPT-5 in the malignancy discrimination task on the ISIC dataset (melanoma vs. benign lesions). GPT-5 achieved an area under the curve (AUC) of 0.672, indicating a moderate ability to distinguish malignant from benign lesions.

To more comprehensively evaluate GPT-5 performance, in addition to sensitivity, specificity, accuracy, and F1 score, we computed Cohen’s kappa to assess agreement between GPT-5 predictions and ground truth beyond chance expectations. We also generated receiver operating characteristic (ROC) curves and corresponding area under the curve (AUC) values to quantify the model’s ability to discriminate malignant from benign lesions across decision thresholds. An example ROC curve is shown in Figure A3 (Appendix A.3), and kappa and AUC results for the top-1 diagnosis and malignancy discrimination tasks are summarized in Table 3.

As shown in Table 3, GPT-5 achieved a Cohen’s kappa of 0.220 and an AUC of 0.535 on ISIC, and a Cohen’s kappa of 0.360 and an AUC of 0.515 on HAM10K for top-1 diagnosis, indicating poor agreement beyond chance and limited discriminatory ability in primary prediction. In contrast, GPT-5 performed better in malignancy discrimination than in top-1 diagnosis, with consistently stronger results on HAM10K than on ISIC. Its highest performance was observed on HAM10K for malignancy discrimination (κ = 0.417, AUC = 0.763), consistent with the trend seen in the other evaluation metrics.

### 3.6. Impact of Sampling Temperature on GPT-5 Performance

Large language models such as GPT-5 rely on probabilistic sampling to generate outputs, with the temperature parameter (ranging from 0 to 2) controlling the degree of randomness. This study used GPT-5’s default temperature of 1.0. A higher temperature (e.g., 1.0) produces more diverse responses, whereas a lower temperature (e.g., 0) makes the model more deterministic, consistently selecting the most likely answer. In diagnostic applications like melanoma detection, it is important to determine whether variability introduced by temperature enhances or compromises reliability. To this end, we tested GPT-5 for malignancy discrimination under both temperature settings.

Table 4 summarizes GPT-5’s performance for malignancy discrimination on HAM10K at two temperature settings. Compared with the results at temperature 1.0, performance at temperature 0 was nearly identical (sensitivity 64.8%, specificity 76.0%, accuracy 70.4%, F1 score 68.6%), indicating that lowering the temperature did not improve clinical outcomes in this context. Table 4 only presents the results on HAM10K, as GPT-5 performance on ISIC was unchanged across temperature settings.

It is worth noting that our temperature assessment was limited to malignancy discrimination, as a temperature of 0 produces deterministic outputs without alternative predictions and therefore cannot be applied to the top-3 diagnoses task, which requires response diversity.

## 4. Discussion

This paper used only images to evaluate GPT-5 accuracy for melanoma detection. However, clinical decision-making is inherently multimodal, drawing on textual, visual, and sometimes auditory information. A recent preliminary study showed that combining photographs with textual input improved GPT-4V’s diagnostic accuracy compared with image-only assessment [24]. This finding suggests that the unimodal evaluation presented here may underestimate the full diagnostic potential of GPT-5. Future research incorporating multimodal clinical data will therefore be essential to more comprehensively assess GPT-5’s utility in real-world healthcare settings.

In addition to unimodal assessment, three other limitations should also be noted. First, our reliance on public, well-studied cases may not accurately reflect bona fide diagnostic capability: because GPT-5 was trained on large-scale internet data, some images may overlap with GPT-5’s training data, potentially inflating performance estimates. Second, the study did not include a direct comparison with human expert raters, which may be critical for contextualizing the model’s performance in clinical practice. Third, we did not examine model stability across alternative prompting strategies, factors known to influence LLM behavior.

Furthermore, it is important to recognize that GPT-5, as a general-purpose LLM, is not specifically optimized for medical decision-making. As highlighted in recent evaluations across multiple medical subfields [40], generalist LLMs often lack the domain specificity, factual grounding, and consistency required for reliable clinical use. Enhancing performance for specialized tasks such as melanoma diagnosis will likely require domain adaptation strategies, including retrieval-augmented generation (RAG), melanoma-specific fine-tuning, carefully structured prompting frameworks, and the integration of a curated dermatology knowledge base. These techniques are expected to reduce hallucinations, improve clinical reasoning, and enable more consistent recognition of melanoma-specific visual patterns.

Finally, despite GPT-5’s strong performance in certain tasks, LLM-based systems must be viewed as adjunctive decision-support tools rather than autonomous diagnostic engines. High-quality care is grounded in the principles of evidence-based medicine (EBM), which integrate imaging-based findings with patient history, lesion evolution, clinical examination, and clinician expertise. Accordingly, LLM-generated outputs should complement—not replace—physician judgment. This EBM perspective was the reason why we reported malignancy discrimination and top-3 diagnostic performance, alongside the top-1 diagnosis: while top-1 accuracy evaluates GPT-5’s potential as a primary diagnostic tool, top-3 performance more closely mirrors the process of differential diagnosis in clinical practice.

## 5. Conclusions

In this study, we evaluated GPT-5’s performance in melanoma detection using dermoscopic images from two benchmark datasets, ISIC and HAM10K. GPT-5 achieved moderate accuracy in top-1 predictions and malignancy discrimination but showed substantial gains in top-3 differential diagnoses, reaching sensitivity >93%, specificity >86%, accuracy ≥92%, and F1 score >91% on both datasets. Moreover, GPT-5 outperformed earlier models, including GPT-4V, GPT-4T, and GPT-4o, by offering a more favorable balance between sensitivity and specificity and demonstrating greater overall reliability in malignancy discrimination. These findings highlight GPT-5’s improvement over prior models and its potential to support clinical decision-making and medical education.

This study also revealed an important limitation: GPT-5 showed a greater tendency to misclassify malignant lesions as benign than the reverse, a bias with potential clinical implications. Addressing this issue through calibration and further refinement will be essential before clinical integration.

Furthermore, we explored the impact of sampling temperature on GPT-5 performance. The variation in sensitivity and specificity reported in prior studies for GPT-4T and GPT-4o on HAM10K (and for GPT-4V on ISIC) contrasts notably with the performance of GPT-5. Earlier assessments were conducted manually through the default ChatGPT interface, which operates at a temperature of 1.0 that introduces stochastic variability into responses, while our evaluation of GPT-5 was performed programmatically via the OpenAI API, allowing systematic testing at different temperatures, including 0. Because GPT-5 showed consistent performance across these settings, the variability observed in earlier studies seems unlikely to be explained by temperature differences alone and more likely reflects genuine improvements of GPT-5 over its predecessors.

## Figures and Tables

**Table 1:** Detection performance of GPT-5 and GPT-4V for melanoma on ISIC dermoscopic images.

No	Model	Diagnostic Objective	Sensitivity (%)	Specificity (%)	Accuracy (%)	F1 (%) *	Study ID
1	GPT-5	Top 1 diagnosis	46.0	76.0	61.0	54.0	This Study
		Top 3 diagnoses	100	86.2	92.0	91.3	
		Malignancy discrimination	56.0	72.0	64.0	60.9	
2	GPT-4V	Top 1 diagnosis	32.0	40.0	36.0	33.3	Shifai et al. [20]
		Top 3 diagnoses	56.0	53.3	54.7	55.3	
		Malignancy discrimination	46.0	78.0	62.0	54.8	
3	GPT-4V	Top 1 diagnosis	56.9	38.8	48.0	52.2	Liu et al. [38]
		Top 3 diagnoses			78.0		
		Malignancy discrimination	45.1	42.9	44.0	45.1	

* The F1 scores for Shifai et al. [20] and Liu et al. [38] were calculated by us based on the reported data.

**Table 2:** Detection performance of GPT-5 for melanoma compared with GPT-4T and GPT-4o on 500 HAM10K images.

No	Model	Diagnostic Objective	Sensitivity (%)	Specificity (%)	Accuracy (%)	F1 (%)	Study ID
1	GPT-5	Top 1 diagnosis	50.4	85.6	68.0	61.5	This Study
		Top 3 diagnoses	93.2	100	96.6	96.5	
		Malignancy discrimination	65.1	76.8	70.9	69.1	
2	GPT-4T	Malignancy discrimination	76.3	32.9	54.6	62.7	Sattler et al. [21]
	GPT-4o	Malignancy discrimination	96.8	18.4	57.7	69.5	

**Table 3:** Kappa and AUC results of GPT-5 in top-1 diagnosis and melanoma discrimination.

Dataset	Diagnostic Objective	Kappa	AUC
ISIC	Top 1 diagnosis	0.220	0.535
	Malignancy discrimination	0.280	0.672
HAM10K	Top 1 diagnosis	0.360	0.512
	Malignancy discrimination	0.417	0.763

**Table 4:** GPT-5 performance for malignancy discrimination on HAM10K at temperatures 0 and 1.0.

Temperature	Sensitivity (%)	Specificity (%)	Accuracy (%)	Precision (%)	F1 (%)
1.0	65.1%	76.8%	70.9%	73.6%	69.1%
0	64.8%	76.0%	70.4%	73.0%	68.6%

## Data Availability

All dermoscopic images and clinical metadata analyzed in this study are publicly available and can be obtained directly from the ISIC Archive at https://www.isic-archive.com (assessed on 28 September 2025). Both ISIC and HAM10K data are open for research use, and no proprietary or patient-identifiable data were included. Additionally, all Python scripts developed for this study, including code for downloading and processing images from ISIC and HAM10K, as well as code for querying the OpenAI API, are publicly available at https://github.com/qwangmsk/Melanoma-Detect (accessed on 28 September 2025).

## Abbreviations

The following abbreviations are used in this manuscript:

AUC	Area under the curve
CNN	Convolutional neural network
DL	Deep learning
EBM	Evidence-Based Medicine
GPT	Generative Pre-trained Transformer
HAM10K	Human Against Machine with 10,000 training images
ISIC	International Skin Imaging Collaboration
JSON	JavaScript Object Notation
LLM	Large language model
ML	Machine learning
ROC	Receiver operating characteristic
